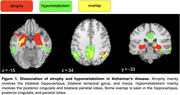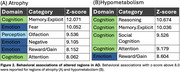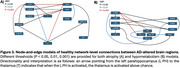# Dissociation of hypometabolism and atrophy and implications on biomarker use in Alzheimer’s disease

**DOI:** 10.1002/alz70862_109972

**Published:** 2025-12-23

**Authors:** Annie Dang, Di Wang, Mohamad Habes, Peter T Fox

**Affiliations:** ^1^ UT Health San Antonio, San Antonio, TX USA; ^2^ University of Texas Health San Antonio, San Antonio, TX USA

## Abstract

**Background:**

Hypometabolism in AD involves the posterior cingulate cortex and temporoparietal areas. Atrophy involves the medial and lateral temporal areas. This discrepancy in hypometabolism and atrophy patterns was first noted in 2002 and incorporated into AD diagnostic criteria in 2011. However, the distinction between hypometabolism and atrophy was lost under the 2016 A/T/N framework, which grouped both together under the “N” category. Iterations of the A/T/N framework (2018, 2024) have added increasing nuance to biomarker profiling. The 2024 version distinguishes between fluid and imaging biomarkers and incorporates quantitative and topological measures available with imaging. The present project provides a timely large‐scale characterization of the dissociation of hypometabolism and atrophy in AD and encourages discussion of incorporation of this phenomenon into the AD biomarker framework. Here, we conduct large‐scale meta‐analyses to characterize atrophy and hypometabolism patterns, compare their behavioral associations, and explore their network‐level connections.

**Method:**

BrainMap databases (voxel‐based morphometry – VBM; voxel‐based physiology – VBP) were used to identify AD studies for meta‐analysis. 412 VBM (atrophy) and 462 VBP (hypometabolism) contrasts were identified. Activation likelihood estimation was performed separately for VBM and VBP to identify cross‐study convergence of brain alteration patterns. Mango was used to visualize results, quantify VBM/VBP spatial overlap, and determine behavioral associations. To explore network‐level connections, meta‐analytic connectivity modeling was performed. VBM/VBP identified regions served as nodes. Edges were identified using task‐activation data from BrainMap and represent healthy functional connectivity between nodes.

**Result:**

VBM and VBP analysis showed different patterns of change. VBM changes predominantly affect the bilateral hippocampus, bilateral temporal gyrus, and insula. VBP changes predominantly affect the posterior cingulate and bilateral parietal lobes (Figure 1). Behavioral analysis showed VBM regions loading most strongly on explicit memory and emotion. VBP regions loaded most strongly on reasoning, explicit memory, and social cognition (Figure 2). Both VBM and VBP alterations occurred in network‐based patterns (Figure 3).

**Conclusion:**

Atrophy and hypometabolism show distinct patterns of alteration in AD, aligning with different behavioral changes. This dissociation suggests that atrophy and hypometabolism may have distinct underlying neuropathologies. We advocate for the inclusion of atrophy and hypometabolism as separate biomarker categories in AD biomarker profiling.